# Obesity- and gender-dependent role of endogenous somatostatin and cortistatin in the regulation of endocrine and metabolic homeostasis in mice

**DOI:** 10.1038/srep37992

**Published:** 2016-11-30

**Authors:** Raúl M. Luque, José Cordoba-Chacon, Ana I. Pozo-Salas, Begoña Porteiro, Luis de Lecea, Rubén Nogueiras, Manuel D. Gahete, Justo P. Castaño

**Affiliations:** 1 Maimonides Institute of Biomedical Research of Cordoba (IMIBIC), Córdoba, Spain; 2 Department of Cell Biology, Physiology and Immunology, University of Córdoba, Córdoba, Spain; 3 Hospital Universitario Reina Sofía (HURS), Córdoba, Spain; 4 CIBER de la Fisiopatología de la Obesidad y Nutrición (CIBERobn), Córdoba, Spain; 5 Campus de Excelencia Internacional Agroalimentario (ceiA3), Córdoba, Spain; 6 Department of Medicine, Section of Endocrinology, Diabetes and Metabolism, University of Illinois at Chicago and Jesse Brown Veteran Affairs Medical Center, Research and Development Division, Chicago, IL, USA; 7 Department of Physiology, CIMUS, University of Santiago de Compostela-Instituto de Investigación Sanitaria, Santiago de Compostela, Spain; 8 Department of Psychiatry and Behavioral Sciences, Stanford University School of Medicine, Palo Alto, CA, USA

## Abstract

Somatostatin (SST) and cortistatin (CORT) regulate numerous endocrine secretions and their absence [knockout (KO)-models] causes important endocrine-metabolic alterations, including pituitary dysregulations. We have demonstrated that the metabolic phenotype of single or combined SST/CORT KO-models is not drastically altered under normal conditions. However, the biological actions of SST/CORT are conditioned by the metabolic-status (e.g. obesity). Therefore, we used male/female SST- and CORT-KO mice fed low-fat (LF) or high-fat (HF) diet to explore the interplay between SST/CORT and obesity in the control of relevant pituitary-axes and whole-body metabolism. Our results showed that the SST/CORT role in the control of GH/prolactin secretions is maintained under LF- and HF-diet conditions as SST-KOs presented higher GH/prolactin-levels, while CORT-KOs displayed higher GH- and lower prolactin-levels than controls under both diets. Moreover, the impact of lack of SST/CORT on the metabolic-function was gender- and diet-dependent. Particularly, SST-KOs were more sensitive to HF-diet, exhibiting altered growth and body-composition (fat/lean percentage) and impaired glucose/insulin-metabolism, especially in males. Conversely, only males CORT-KO under LF-diet conditions exhibited significant alterations, displaying higher glucose-levels and insulin-resistance. Altogether, these data demonstrate a tight interplay between SST/CORT-axis and the metabolic status in the control of endocrine/metabolic functions and unveil a clear dissociation of SST/CORT roles.

Somatostatin (SST) and cortistatin (CORT) are two closely related neuropeptides that share high structural, pharmacological and functional similarities^1^,^2^. SST^3^ is a pleiotropic neuropeptide widely distributed throughout the organism, which regulates a plethora of physiological functions, including inhibition of basal and stimulated secretion from endocrine and exocrine cells, inhibition of gastrointestinal motility, modulation of neurotransmission, metabolism and immune function, as well as inhibition of cell proliferation and differentiation^1^,^3^,^4^,^5^,^6^. In contrast, CORT^7^ is mainly produced in the cerebral cortex and was originally identified for its role in the modulation of sleep cycles, neuronal activity and immune system^7^,^8^,^9^,^10^,^11^. Subsequently, CORT was suggested as an endocrine/metabolic sibling of SST; however, recent evidence demonstrated that CORT is able to trigger unique, and even opposite, endocrine and non-endocrine actions from those exerted by SST, including the regulation of endocrine secretions, the control of immune response or the modulation of neuronal activity^2^,^9^,^12^,^13^,^14^,^15^,^16^,^17^,^18^,^19^,^20^. These functional differences may have various causes, such as minor amino acid sequence disparities between SST and CORT, dissimilar capacity to bind and/or activate common receptors (sst1–5)^1^ or, to the ability of CORT, but not SST, to bind other non-sst receptors (i.e. ghrelin receptor, GHSR1a, or mas-related G protein coupled receptor MrgX2)^21^,^22^.

SST and CORT play complex regulatory roles within the endocrine-metabolic milieu. Indeed, SST and CORT are crucial regulators of several metabolic hormones such as growth hormone (GH), prolactin or ACTH/costicosterone, and have also been shown to suppress insulin secretion *in vitro*^2^,^23^, strongly suggesting a relevant role in the modulation of whole-body metabolic function. Accordingly, previous studies using genetically engineered preclinical mouse models have shown that lack of endogenous SST or CORT drastically, but distinctly, alters the normal hormone secretion patterns. Indeed, SST-knockout (KO) mice exhibit gender-dependent elevated levels of GH, prolactin and ACTH/corticosterone, while CORT-KO mice also present gender-dependent elevated levels of GH and ACTH/corticosterone but reduced levels of prolactin^13^,^24^,^25^,^26^,^27^. In addition, although male and female SST-KO mice exhibit comparable glucose/insulin sensitivity to WT controls^13^,^28^, male CORT-KO mice present a clear insulin resistance under chow-diet, normal weight conditions^13^, confirming the gender-dependent role of SST/CORT on metabolic function. However, SST and CORT actions might also be dependent on, or influenced by specific endocrine/metabolic conditions, like obesity^13^,^24^,^25^,^26^,^27^, which may suggest that the final biological actions of SST and CORT may be conditioned by the specific metabolic status. Therefore, the present study has been conceived to unveil, for the first time, the putative interplay between SST or CORT and an extreme metabolic situation such as diet-induced obesity in the control of the most relevant pituitary hormonal axes and the whole-body metabolic function by using male and female SST- and CORT-KO mice fed a low-fat or a high-fat diet and, their respective lean and obese littermate controls.

## Results

### SST and CORT exerted gender- and diet-dependent effects on BW

**Figure 1:**
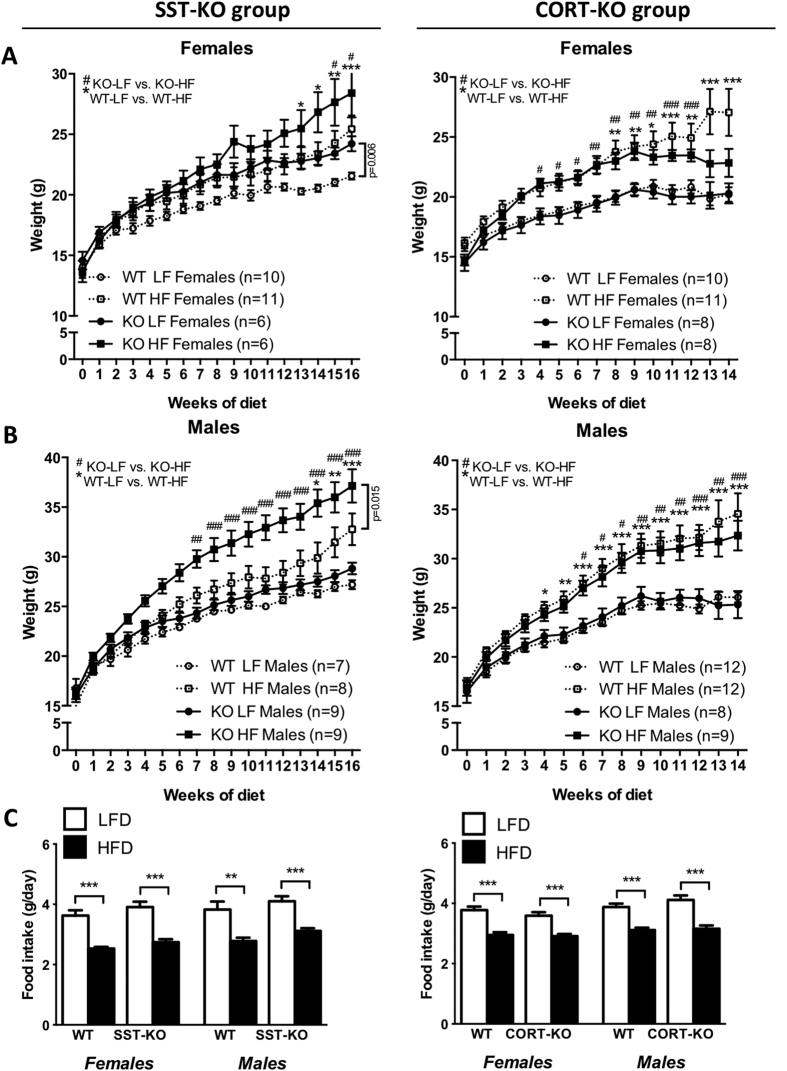
Growth rate and food intake of male and female, LF- and HF-fed, SST-KO, CORT-KO and control mice. Female (**A**) and male (**B**) SST-KO, CORT-KO and control (WT) mice were fed a LF- of a HF-diet starting at 4 weeks of age and body weights were recorder weekly until sacrifice (14–16 weeks of diet). Statistical differences were assessed by two-way ANOVA within genotype and gender followed by Bonferroni post-hoc test within each time point [significant differences are indicated by *(WT-LF vs. WT-HF) or ^#^(KO-LF vs. KO-HF)]. (**C**) Food intake was estimated at 15–17 weeks of age (11–13 weeks of diet) by daily weighting food provided and withdrawn during 7 consecutive days. Statistical differences were assessed by two-way ANOVA within genotype and gender followed by Bonferroni post-hoc test [significant differences between LF- and HF-fed mice are indicated by asterisks (***p < 0.001; **p < 0.01; *p < 0.05). Data represent MEM ± SEM of n = 6–12 mice per gender, diet and genotype.

To unveil the putative interplay between SST or CORT and obesity, we firstly analyzed BW gain and body composition in male and female SST-KO, CORT-KO and their littermate WT-controls fed a LF- or a HF-diet. Under LF-conditions, SST-KO females exhibited significantly higher BW gain (p = 0.006, two-way ANOVA), while male SST-KO or CORT-KO males and females did not show significant differences with WT-controls (Fig. 1A,B). As expected, HF-feeding significantly increased BW gain in all experimental groups (p < 0.05, two-way ANOVA) (Fig. 1A,B). Under HF-diet conditions, male SST-KO exhibited increased BW gain compared to controls (Fig. 1B), while female SST-KO and CORT-KO male and female did not show differences (Fig. 1A,B).

The increase in BW observed in SST-KO mice, especially in males, was not associated to increased calories intake. Indeed, although HF-fed animals exhibited reduced food intake compared to genotype- and gender-matched LF-fed mice, SST-KO and CORT-KO mice exhibited similar food intake than their controls within the same diet (Fig. 1C).

**Figure 2:**
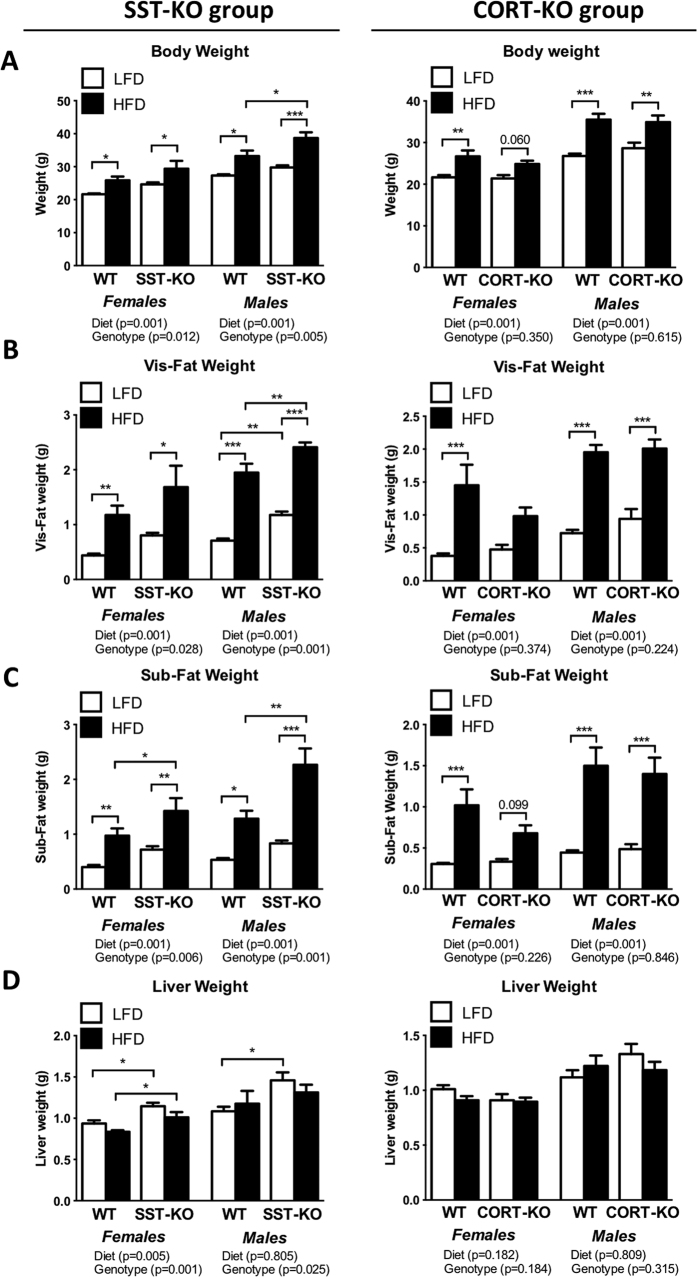
Body, visceral and subcutaneous fat depots and liver weights of male and female, LF- and HF-fed, SST-KO, CORT-KO and control mice. Body (**A**), visceral (**B**) and subcutaneous (**C**) fat depots and liver (**D**) weights of female and male SST-KO, CORT-KO and control (WT) mice fed a LF- of a HF-diet were recorder at sacrifice (14–16 weeks of diet). Statistical differences were assessed by two-way ANOVA within genotype and gender (indicated below each graph) followed by Bonferroni post-hoc test [significant differences between LF- and HF-fed or between WT and KO mice are indicated by asterisks (***p<0.001; **p < 0.01; *p < 0.05)]. Data represent MEM ± SEM of n = 6–12 mice per gender, diet and genotype.

The predominant effect of SST in the control of BW gain was further supported by the BWs at sacrifice (Fig. 2A). Specifically, HF-diet induced significant BW increases in all the experimental groups compared to LF-fed counterparts (p < 0.001, two-way ANOVA) (Fig. 2A). However, only SST-KO mice exhibited significant difference on BW compared to WT-controls (p = 0.012 and p = 0.005 two-way ANOVA in females and males, respectively), being significantly marked in HF-fed males (p < 0.05, Bonferroni test; Fig. 2A). No significant changes were observed in CORT-KO mice compared to WT-controls (Fig. 2A).

### SST and CORT exerted gender- and diet-dependent effects on body composition

HF-diet feeding increased visceral and subcutaneous fat depots weight in most of the experimental groups compared to LF-diet controls (p < 0.001, two-way ANOVA within genotype and gender; Fig. 2B,C). Indeed, circulating leptin levels were elevated in all the HF-diet fed groups compared to LF-fed mice (p < 0.05, two-way ANOVA within genotype and gender; Supplemental Fig. 2A). However, only lack of endogenous SST elicited significant changes in fat depot weight (Fig. 2B,C), exhibiting a significant increment on visceral and subcutaneous fat depots weight irrespective of gender (p < 0.001, two-way ANOVA), while lack of CORT did not exert any significant effect. These changes were mostly observed in SST-KO males (Fig. 2B,C) and were particularly pronounced in the subcutaneous adipose depot of HF-diet fed SST-KO males (1.8-fold; p < 0.001, Bonferroni test).

Similarly, liver weights were altered by the absence of SST but not CORT. Indeed, SST-KO females and males exhibited increased liver weights (p < 0.001 and p = 0.025, two-way ANOVA, respectively), being more pronounced in females, irrespective of the diet, and in LF-diet males (Fig. 2D). In contrast, no significant changes in pancreas weight were found in any experimental groups (Supplemental Fig. 2B).

### Lack of SST and CORT altered the production and secretion of pituitary hormones

**Figure 3:**
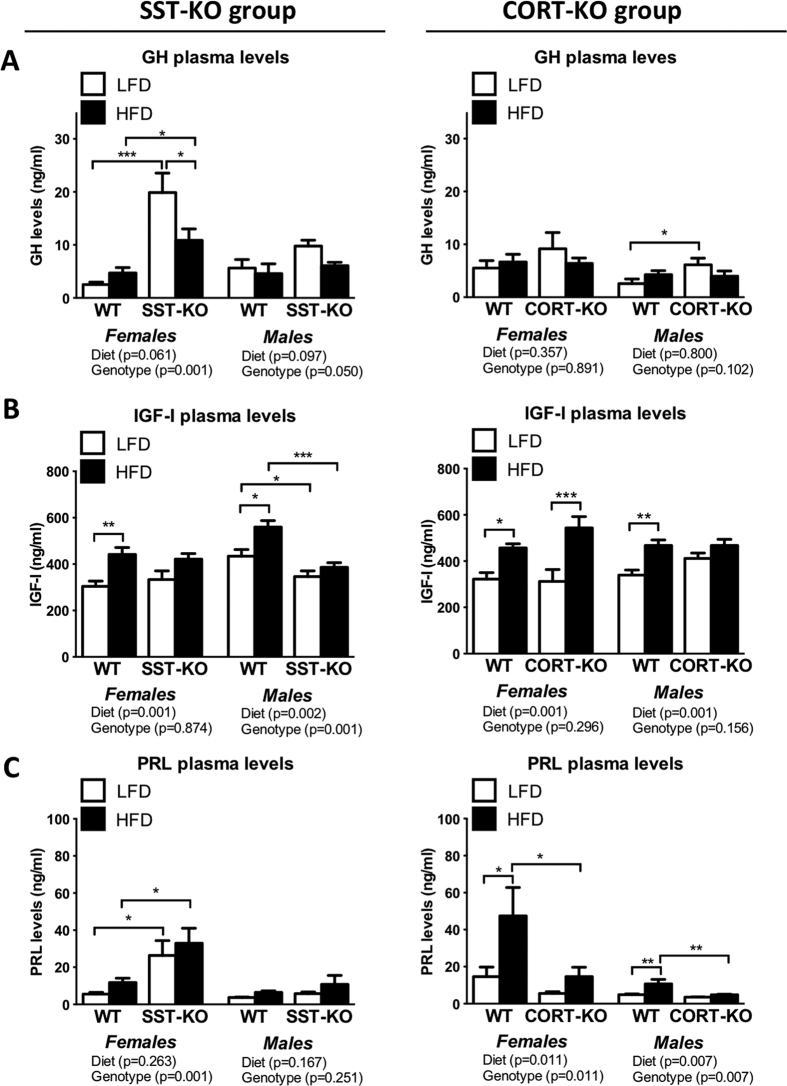
GH, IGF-I and prolactin (PRL) levels of male and female, LF- and HF-fed, SST-KO, CORT-KO and control mice. GH (**A**), IGF-I (**B**) and PRL (**C**) levels of female and male SST-KO, CORT-KO and control (WT) mice fed a LF- of a HF-diet determined at sacrifice (14–16 weeks of diet). Statistical differences were assessed by two-way ANOVA within genotype and gender (indicated below each graph) followed by Bonferroni post-hoc test [significant differences between LF- and HF-fed or between WT and KO mice are indicated by asterisks (***p < 0.001; **p < 0.01; *p < 0.05)]. Data represent MEM ± SEM of n = 6–12 mice per gender, diet and genotype.

We analyzed the interaction of diet-induced obesity with the lack of SST and CORT on the production and secretion of key pituitary hormones by determining GH, IGF and PRL levels at sacrifice (Fig. 3). GH is a pivotal metabolic hormone secreted in a pulsatile fashion and alterations in GH secretion pattern are difficult to identify by simple point bleeding. However, our measurements demonstrated a significant effect of the genotype on GH levels of SST-KO females and males (p = 0.001 and p = 0.05, two-way ANOVA, respectively), being especially significant in SST-KO females (p < 0.001 and p < 0.05, Bonferroni test in LF- and HF-fed, respectively; Fig. 3A). However, these changes cannot be explained by increased expression of pituitary GH mRNA (Supplemental Fig. 3A), which suggests the existence of alternative mechanism in SST-KO mice. In contrast, and likely due to the reasons mentioned above, only significant increases in GH levels were found in LF-fed CORT-KO males compared with controls (Fig. 3A); while differences in other CORT-KO mice groups were not apparent. Similarly, no changes in pituitary GH mRNA levels in CORT-KO mice were found (Supplemental Fig. 3A). Remarkably, changes in GH levels could not be explained by alterations in the expression of hypothalamic factors (GHRH or ghrelin, Supplemental Fig. 3C,D); however, since we analyzed the expression levels of the whole hypothalamus, local changes in specific hypothalamic nuclei cannot be discarded.

Furthermore, although no significant changes were found in CORT-KOs compared to controls (Supplemental Fig. 4B,C), SST-KO mice showed drastic alterations in the hepatic expression of MUP-3 and PRL-R, two reliable markers of altered GH pulsatile pattern. Particularly, female LF-fed SST-KO mice presented higher levels of PRL-R, while female HF-fed SST-KO mice showed reduced levels of MUP-3 expression compared to diet-matched controls (Supplemental Fig. 4B,C), suggesting altered GH secretion patterns. The most striking results were obtained in SST-KO males, which exhibited significantly elevated levels of PRL-R and reduced levels of MUP-3 compared to controls (Supplemental Fig. 4B,C), indicating a drastically feminized GH pattern.

Similarly, HF-feeding seemed to induce a significant elevation in IGF-I levels in SST-KO and CORT-KO mice (p < 0.001, two-way ANOVA), which was specially pronounced in controls (Fig. 3B). Although CORT-KO mice exhibited similar IGF-I levels than controls, SST-KO male mice showed reduced IGF-I levels, irrespective of the diet (p = 0.001, two-way ANOVA; Fig. 3B). Interestingly, the circulating pattern of IGF-I was similar to the hepatic expression pattern of IGF-I in SST-KO mice, although no significant differences were detected in this cohort (Supplemental Fig. 4A). No changes were observed in the hepatic expression of IGF-I of CORT-KO mice.

Inasmuch as we have previously reported different roles of SST and CORT in the control of PRL levels^13^, we sought to determine the putative interaction between SST or CORT and diet-induced obesity in PRL. Consistently, SST-KO mice exhibited similar PRL level elevations under both dietary conditions (Fig. 3C), being especially drastic in females (p = 0.001, two-way ANOVA) irrespective of diet (Fig. 3C). In addition, PRL levels were significantly reduced in CORT-KO mice (effect of genotype: p < 0.001, two-way ANOVA), being these changes especially marked in HF-diet mice (p < 0.001 and p < 0.05, Bonferroni test in HF-fed females and males, respectively) compared to the gender-matched controls (Fig. 3C). In both cases, changes in circulating PRL levels were not clearly associated to altered pituitary PRL expression of SST-KO and CORT-KO mice, except in the case of female SST-KO wherein PRL expression at the pituitary levels were increased in LF-fed SST-KO and HF-fed controls compared to LF-fed controls (Supplemental Fig. 3B).

### SST and CORT exerted gender- and diet-dependent effects on glucose/insulin homeostasis

**Figure 4:**
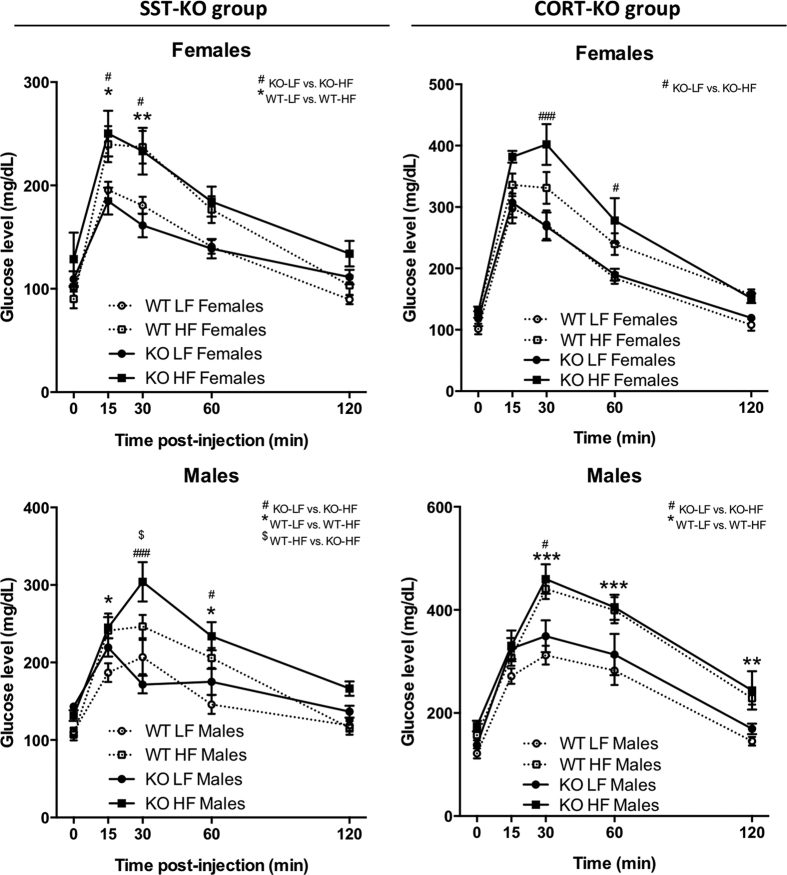
Dynamic glucose tolerance test on male and female, LF- and HF-fed, SST-KO, CORT-KO and control mice. Glucose tolerance tests (GTTs) were performed at 16–18 weeks of age (12–14 weeks of diet) after an overnight fast (2 g/kg glucose, ip) on female and male SST-KO, CORT-KO and control (WT) mice. Statistical differences were assessed by two-way ANOVA within genotype and gender followed by Bonferroni post-hoc test within each time point [significant differences are indicated by *(WT-LF vs. WT-HF), ^#^(KO-LF vs. KO-HF) or ^$^(WT-HF vs. KO-HF)]. Data represent MEM ± SEM of n = 6–12 mice per gender, diet and genotype.

To explore the role of SST and CORT in the interaction between diet and glucose/insulin homeostasis, SST-KO and CORT-KO mice were subjected to *in vivo* dynamic insulin/glucose sensitivity tests. HF-diet feeding induced a drastic, significant impairment of glucose clearance during the GTT, which was observed in all the experimental groups (Fig. 4). Of note, lack of CORT did not impact glucose clearance. On the other hand, although female SST-KO mice exhibited similar GTT curves than WT-controls, male SST-KO mice fed a HF-diet showed impaired glucose clearance compared to controls (Fig. 4), while these differences were not observed in LF-fed mice. These results were further confirmed by the analysis of the area under the curves (AUC) calculated from the GTTs, which showed a significant effect of the diet (p < 0.001, two-way ANOVA) and of genotype in SST-KO males (p = 0.049, two-way ANOVA) (Supplemental Fig. 5). These altered GTT curves were accompanied by changes in fasting, but not fed, glucose levels in SST-KO males (Supplemental Figure 6A). Of note, no alterations in fed glucose were observed in SST-KO and CORT-KO groups (Supplemental Figure 6B). Similarly, fed insulin levels were altered in SST-KO males (p = 0.012, two-way ANOVA), being significantly elevated in SST-KO HF-fed males compared to HF-fed controls and LF-fed SST-KO mice; while no alterations were observed in other experimental groups (Supplemental Figure 6C).

**Figure 5:**
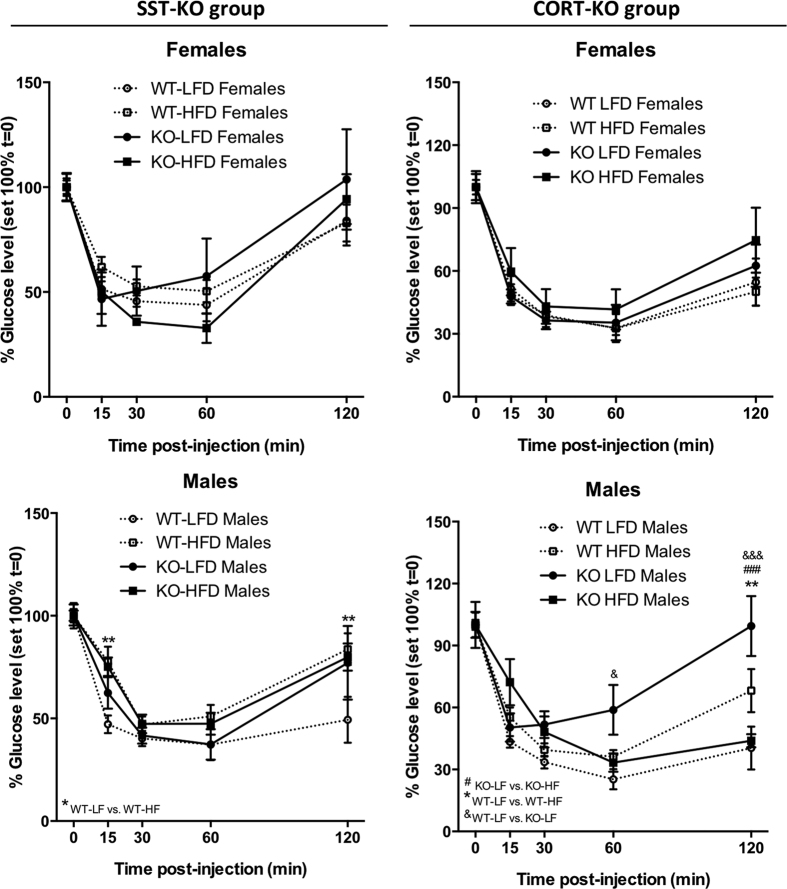
Dynamic insulin tolerance test on male and female, LF- and HF-fed, SST-KO, CORT-KO and control mice. Insulin tolerance tests (ITTs) were performed at 17–19 weeks of age (13–15 weeks of diet) under ad libitum fed conditions (1 U/kg Novolin, ip) on female and male SST-KO, CORT-KO and control (WT) mice. Statistical differences were assessed by two-way ANOVA within genotype and gender followed by Bonferroni post-hoc test within each time point [significant differences are indicated by *(WT-LF vs. WT-HF), ^#^(KO-LF vs. KO-HF) or ^&^(WT-LF vs. KO-LF)]. Data represent MEM ± SEM of n = 6–12 mice per gender, diet and genotype.

Implementation of ITTs served to confirm a certain degree of insulin resistance of HF-fed WT males but not females, mainly indicated by a lower recovery of glucose levels at 120 minutes post-injection (Fig. 5). Interestingly, LF-fed CORT-KO males were more insulin resistant than LF-fed WT-controls (Fig. 5); while no differences where observed in other experimental groups.

## Discussion

SST and CORT are two neuropeptides widely known by their ability to regulate numerous endocrine secretions, particularly those from the pituitary gland (GH or PRL) and the gastrointestinal tract (pancreatic and gut secretions)^2^. Indeed, although they exert different, even opposite effects in the regulation of several non-endocrine functions, both peptides seem to similarly regulate endocrine secretions^2^, with the only reported exception of PRL^13^. The fact that both peptides exhibit a marked inhibitory action on GH release and other endocrine secretions suggests that lack of SST or CORT would cause profound alterations in relevant endocrine/metabolic processes. However, genetically engineered animal models have revealed that, under normal conditions, the single or combined deletion of SST and/or CORT does not drastically influence the metabolic phenotypic of these animals^13^,^29^,^30^. Indeed, despite the fact that SST and CORT are two primary inhibitors of GH secretion by acting through their canonical receptors (sst1–5)^13^,^26^,^31^,^32^ and, that SST-KO or CORT-KO mice display markedly elevated plasma GH levels^13^,^25^, SST-KO and CORT-KO mice do not exhibit a correspondingly enhanced somatic growth^13^,^25^ or major changes in body mass, IGF plasma levels or glucose/insulin metabolism^13^,^24^,^25^,^29^,^30^,^32^.

However, SST and CORT actions are clearly dependent on, and influenced by, the endocrine/metabolic situation, such as fasting^25^,^27^ and/or obesity^13^,^24^,^25^,^26^,^27^,^33^, which suggest that the final biological actions of SST and CORT may be conditioned by the specific metabolic status. For this reason, in this study, we used male and female SST- and CORT-KO mice fed a LF or a HF diet to thoroughly explore, for first time, the interplay between SST or CORT and obesity in the control of the most relevant pituitary hormonal axes and whole-body metabolic function (the main results are summarized in Table 1) and found striking differences that may help to shed light to the role of endogenous SST and CORT in the control of the endocrine/metabolic milieu under extreme metabolic conditions.

As expected, SST-KO and CORT-KO mice exhibited drastic deregulations in key pituitary axes, such as GH and PRL, which were overtly maintained under LF- and HF-conditions. In particular, male and female SST-KO and CORT-KO mice showed elevated plasmatic GH levels irrespective of the diet (except for the case of CORT-KO females), which is consistent with previous reports observing similar changes under normal and extreme metabolic conditions^13^,^25^,^27^,^29^,^34^,^35^. Of note, these changes seemed to be more pronounced under LF conditions, wherein SST-KO and CORT-KO mice exhibited obvious elevated GH levels compared to controls. The fact that these changes were less evident under HF-feeding could be associated to the fact that obesity represents an extreme metabolic status that courses with clear impairments in GH pulses^36^,^37^, which could be masking the loss of SST/CORT inhibitory effect. Remarkably, these changes in plasma GH levels were not accompanied by parallel alterations in the expression of pituitary GH mRNA, which is consistent with previous reports^25^,^27^,^29^,^38^,^39^ and reinforces the idea that SST and CORT could be exerting regulatory roles at different levels of the regulatory axis of the GH secretion, including the hypothalamic level, the control of secretory pulses or the regulation of other hormonal systems (ghrelin, glucocorticoids, etc.) as previously suggested^13^,^25^,^27^,^29^,^30^,^33^. Nevertheless, it is important to highlight that, despite the difficulty of assessing GH levels due to its pulsatile secretion pattern, the results presented herein serve to confirm previous results on the crucial role of endogenous SST and CORT on the control of GH secretion and to expand them to different dietary patterns. Indeed, the analysis of the expression of GH pattern-sensitive genes in the liver of SST-KO under LF- and HF-conditions further reinforced the idea that the GH secretion pattern is dramatically altered by the lack of SST inasmuch as SST-KO male mice exhibited a feminized hepatic expression of PRLR and MUP3 gene [two genes whose expression depends on the GH pulsatile pattern^40^], which is clearly maintained under LF- and HF-feeding, consistent with previous data on male SST-KO fed a chow diet^25^.

Similarly, the effect of SST and CORT on the secretion of PRL was not influenced by the metabolic status since SST-KO mice presented elevated and CORT-KO exhibited reduced PRL levels, irrespective of the diet, which is consistent with the results previously reported by our group under standard feeding conditions^13^. In this case, comparable changes have been found under LF- and HF-conditions, reinforcing the idea that both peptides exert a direct role on the regulation of PRL release at pituitary level and dismissing the putative indirect action through the regulation of other endocrine-metabolic mediators. These results also extend previous data showing an opposite role of SST and CORT on the regulation of PRL release and demonstrate that CORT is not a mere SST analog in the regulation of endocrine secretions, which have been also observed in the case of non-endocrine actions^2^,^9^,^12^,^13^,^14^,^15^,^16^,^17^,^18^,^19^,^20^.

In contrast, despite clear alterations in pituitary axes, lack of SST or CORT did not drastically impacted mice growth, body composition or glucose/insulin metabolism, which is in agreement with previous data reported in single SST-KO or CORT-KO mice or even in double SST/CORT-KO mice under standard dietary conditions^13^,^25^,^29^,^30^. Indeed, although HF-feeding demonstrated a significant effect on all the experimental groups in terms of increased BW gaining, reduced food intake, elevated adipose tissue weight, altered body composition and impaired glucose/insulin metabolism, lack of SST or CORT showed minor effects. Particularly, lack of SST did not significantly influence linear growth, body composition or glucose/insulin homeostasis under LF-diet feeding, which is consistent with previous studies^27^,^28^, but interestingly, augmented the deleterious effects of HF-feeding, especially on male mice. Indeed, HF-fed SST-KO male mice exhibited significantly higher body weight, with non-significant elevation in the BW of HF-fed SST-KO females compared to controls, which could be barely explained by the discrete tendency of the SST-KO animals to exhibit higher food intake. In addition, both male and female SST-KO mice presented altered body composition when fed a HF-diet, with increased visceral and subcutaneous adipose tissue depots and liver weight; changes that were more pronounced in males. Interestingly, male and female SST-KO mice displayed unaltered ITTs under HF-diet, consistent with previous results under standard conditions^13^; however, males presented elevated plasmatic levels of insulin and glucose and impaired glucose tolerance, suggesting that female SST-KO mice could be, somehow, protected, at least partially, from the deleterious effects of HF-feeding^41^. These data are also consistent with previous studies under standard conditions^13^ which, altogether, indicate that SST is a key inhibitory regulator of weight gain, body composition and whole body glucose homeostasis under conditions of normal/high, but not low, calorie intake.

In contrast to SST-KO mice, lack of endogenous CORT seems to be more important under LF-feeding conditions than in diet-induced obesity conditions. Indeed, although female CORT-KO exhibited a similar phenotype than control mice under LF-diet, male CORT-KO mice displayed impaired ITT and elevated fasting glucose levels, consistent with previously reports^13^. These data reinforce the idea that CORT-KO mice are insulin resistant but glucose tolerant in conditions of low/moderate calorie intake, which could be associated to a particular pattern and/or magnitude of GH release in CORT-KO mice that could modify the hepatic glucose production in response to hyperglycemia or to concomitant changes in other regulatory systems such as glucocorticoids^42^ or ghrelin^24^,^25^,^43^,^44^,^45^ as previously hypothesized^13^. However, lack of CORT was not essential to maintain linear growth, body composition or appropriate glucose/insulin metabolism under high calories intake as HF-fed CORT-KO males and females exhibited similar metabolic alterations than HF-fed controls.

Interestingly, we have recently reported that CORT is essential to protect the mammary gland against the chemical-induced tumorigenesis and that this action is diet-independent, while the role of SST seems to be more restricted to HF-conditions^33^, which suggests a tight interplay between the SST/CORT axis and the metabolic (lean/obese) status in the control of a wide variety of physiological and pathological functions that may strongly depend on the particular end-point analyzed and, therefore, highlight the necessity of taking into consideration this interplay when analyzing the role of these pivotal hormones. Altogether, the data presented herein demonstrate a gender- and diet-dependent role of endogenous SST and CORT on the control of key endocrine and metabolic parameters such as pituitary hormones secretion (i.e. GH and PRL), body growth and body composition or glucose/insulin homeostasis and, add further evidence to the contention that SST and CORT are not two mere endocrine functional siblings. Indeed, in this case, SST seems to be more important than CORT in controlling the parameters mentioned above and, therefore, suggests a clear dissociation of SST/CORT physiological functions. Therefore, additional efforts should be needed to unveil the specific and distinctive mechanism of action of SST and CORT, inasmuch as this novel information could pave the way towards the identification and/or development of useful tools in the management of endocrine-metabolic pathologies.

## Material and Methods

### Animal studies

All experimental procedures were approved by the IACUC of the University of Cordoba and were performed in accordance with the appropriate guidelines and regulations. C57Bl6/J SST-KO (kindly provided by Dr. Ute Hochgeschwender) and CORT-KO mice were bred in-house and maintained under standard conditions (12 h light/dark cycle; lights on at 07:00 h; 22–24C), with free access to tap water and food [standard rodent chow (SAFE-diets, Barcelona, Spain)]. The development and validation of Sst−/− and Cort−/− mice as well as the methods to determine the genotypes by conventional PCR of tail-snip DNA have been previously reported^13^,^27^,^30^,^33^,^46^. Specifically, male and female C57Bl/6 J wild-type (Sst +/+ and Cort +/+; Controls, WT), SST-KO (Sst −/−) and CORT-KO (Cort −/−) littermate mice were generated from heterozygous breeding pairs (Sst+/− or Cort +/− mice, respectively). Littermates were singled-housed at 4 weeks of age and randomly divided in two groups fed a low-fat (LF; Research Diets, Gentofte, Denmark; D12450B; 10% Kcal fat, 70% Kcal carbohydrates, 20% Kcal proteins) or a micronutrient-matched high-fat diet (HF; Research Diets; D12492; 60% Kcal fat, 20% Kcal carbohydrates, 20% Kcal proteins) for 14–16 weeks (n = 6–12 mice per gender, diet and genotype). Before experimental manipulation and/or euthanasia, mice were handled daily for two weeks to acclimate them to personnel and handling methods. All (male or female) mice were euthanized the same day (between 08:00–10:00 h) by decapitation without anesthesia and trunk blood was collected, mixed with EDTA and Miniprotease inhibitor (Roche, Barcelona, Spain), and kept in ice until further centrifugation to obtain plasma. All females were euthanized under random cycling conditions. Tissues were immediately excised, weighted and snap-frozen in liquid nitrogen. In particular, visceral and subcutaneous fat depots were directly dissected from the abdominal cavity and the abdominal subcutaneous region, respectively. Plasma and tissues were stored at −80C until posterior analysis. Analysis of SST and CORT levels at the hypothalamus by qPCR (see below) further confirmed the genotypes (Supplemental Fig. 1).

### *In vivo* evaluation of metabolic status

Every week, body weight (BW) was monitored to screen the effectiveness of HF-diet to induce an obese phenotype. Food intake was estimated at 15–17 weeks of age (11–13 weeks of diet) by daily weighting food provided and withdrawn during 7 consecutive days. Glucose tolerance tests (GTTs) were performed at 16–18 weeks of age (12–14 weeks of diet) after an overnight fast (2 g/kg glucose, ip) and insulin tolerance tests (ITTs) were performed at 17–19 weeks of age (13–15 weeks of diet) under ad libitum fed conditions (1 U/kg Novolin, ip), beginning between 0800 h–0900 h. Blood was collected at t0, for insulin and glucose levels determination.

### Circulating hormones measurement

Blood glucose was assessed by glucometer (Accu-Chek system; Roche Diagnostics, Barcelona, Spain). Commercial ELISA kits validated for the *in vitro* quantitative measurement of hormones in mouse serum or plasma were used to assess circulating GH, insulin (Millipore), leptin, IGF-I (Immunodiagnostic Systems, Bolton, UK) and prolactin (CalBiotech, Spring Valley, CA) following manufacturer’s instructions.

### Quantitative real-time PCR (qPCR)

Total RNA from mouse tissues (hypothalamus, pituitary and liver) was extracted, reverse transcribed, and amplified by quantitative real-time PCR using specific sets of primers, as previously described^13^,^30^,^47^,^48^. To control for variations in the amount of RNA used and the efficiency of RT reaction, mRNA copy number of each transcript was adjusted by a normalization factor (NF) obtained from the expression of three housekeeping genes [Beta-actin (ACTB), cyclophilin A and/or Hypoxanthine-guanine phosphoribosyltransferase (HPRT)], using the Genorm 3.3 application^49^, where the expression of these housekeeping genes was not significantly altered between the experimental groups. Details of primers used in qPCR are provided in Suppl. Table 1.

### Statistical analysis

Samples from all groups were processed at the same time. Male and female results were analyzed independently and the effect of genotype, the diet and/or the time was assessed by two-way ANOVA, followed by Bonferroni test for multiple comparisons. All values are expressed as mean ± SEM. p < 0.05 was considered significant. All statistics analyses were performed using the GraphPad Prism 5.0 software (GraphPad Software Inc., La Jolla, CA, USA).

## Additional Information

**How to cite this article**: Luque, R. M. *et al*. Obesity- and gender-dependent role of endogenous somatostatin and cortistatin in the regulation of endocrine and metabolic homeostasis in mice. *Sci. Rep.* **6**, 37992; doi: 10.1038/srep37992 (2016).

**Publisher's note:** Springer Nature remains neutral with regard to jurisdictional claims in published maps and institutional affiliations.

## Supplementary Material

Supplementary Information

## Figures and Tables

**Table 1:** Summary of the endocrine-metabolic parameters determined in female and male SST-KO, CORT-KO and control (WT) mice fed a LF- of a HF-diet.

	SST-KO				CORT-KO			
	Females		Males		Females		Males	
	LFD	HFD	LFD	HFD	LFD	HFD	LFD	HFD
Body weight gain	**↑**	**=**	**=**	**↑**	**=**	**=**	**=**	**=**
Food intake	**=**	**=**	**=**	**=**	**=**	**=**	**=**	**=**
Body weight (sacrifice)	**=**	**=**	**=**	**↑**	**=**	**=**	**=**	**=**
Visceral fat weight	**=**	**=**	**↑**	**↑**	**=**	**=**	**=**	**=**
Subcutaneous fat weight	**=**	**↑**	**=**	**↑**	**=**	**=**	**=**	**=**
Plasma Leptin levels	**=**	**=**	**=**	**=**	**=**	**=**	**=**	**=**
Liver weight	**↑**	**↑**	**↑**	**=**	**=**	**=**	**=**	**=**
Pancreas weight	**=**	**=**	**=**	**=**	**=**	**=**	**=**	**=**
Plasma GH levels	**↑**	**↑**	**=**	**=**	**=**	**=**	**↑**	**=**
Pituitary GH expression	**=**	**=**	**=**	**=**	**=**	**=**	**=**	**=**
Hypothalamic Ghrelin expression	**=**	**=**	**=**	**=**	**=**	**=**	**=**	**=**
Hypothalamic GHRH expression	**=**	**=**	**=**	**=**	**=**	**=**	**=**	**=**
Liver MUP3 expression	**=**	**↓**	**↓**	**↓**	**=**	**=**	**=**	**=**
Liver PRL-R expression	**↑**	**=**	**↑**	**↑**	**=**	**=**	**=**	**=**
Plasma IGF-I levels	**=**	**=**	**↓**	**↓**	**=**	**=**	**=**	**=**
Liver IGF-I exxpressiion	**=**	**=**	**=**	**=**	**=**	**=**	**=**	**=**
Plasma PRL levels	**↑**	**↑**	**=**	**=**	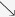	**↓**	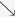	**↓**
Pituitary PRL expression	**=**	**↑**	**=**	**=**	**=**	**=**	**=**	**=**
GTT	**=**	**=**	**=**	**↓**	**=**	**=**	**=**	**=**
AUC GTT	**=**	**=**	**=**	**=**	**=**	**=**	**=**	**=**
ITT	**=**	**=**	**=**	**=**	**=**	**=**	**↓**	**=**
AUC ITT	**=**	**=**	**=**	**=**	**=**	**=**	**=**	**=**
Fasting Glucose levels	**=**	**=**	**↑**	**↑**	**=**	**=**	**=**	**=**
Fed Glucose levels	**=**	**=**	**=**	**=**	**=**	**=**	**=**	**=**
Fed Insulin levels	**=**	**=**	**=**	**↑**	**=**	**=**	**=**	**=**

**↑** and **↓** indicate significant changes compared to sex and diet matched wild-type controls.

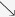
 indicate non-significant tendencies compared to sex and diet matched wild-type controls.
