# Supplementary material for: Obesity- and gender-dependent role of endogenous somatostatin and cortistatin in the regulation of endocrine and metabolic homeostasis in mice
Source: Sci Rep. 2016 Nov 30;6:37992. doi: 10.1038/srep37992 (PMC5128804; doi:10.1038/srep37992)
Supplement: Supplementary Information [file srep37992-s1.pdf]

SUPPLEMENTARY INFORMATION

Obesity- and gender-dependent role of endogenous somatostatin and cortistatin in the regulation of endocrine and metabolic homeostasis in mice

Raúl M. Luque, José Cordoba-Chacon, Ana I. Pozo-Salas, Begoña Porteiro, Luis de Lecea, Rubén Nogueiras, Manuel D. Gahete, Justo P. Castaño

Supplemental Table 1.- Primer used to measure transcript expression by qPCR

Transcript name	Accession number	Primer Sn	Primer As	Product size
GH	NM_008117	CCTCAGCAGGATTTTCACCA	CTTGAGGATCTGCCCAACAC	142
PRL	NM_011164	GGCCATCTTGGAGAAGTGTG	ACAGATTGGCAGAGGCTGAA	140
IGF-I	NM_010512	TCGTCTTCACACCTCTTCTACCT	ACTCATCCACAATGCCTGTCT	202
SST	NM_009215	TCTGCATCGTCCTGGCTTT	CTTGGCCAGTTCCTGTTTCC	113
CORT	NM_007745	AAGAGACCCTCGTCCACCAA	ACCAGGCAAGGAAAGTCAGAAG	213
GHRH	M31654	TGCCATCTTCACCACCAAC	TCATCTGCTTGTCCTCTGTCC	158
Ghrelin	NM_021488	TCCAAGAAGCCACCAGCTAA	AACATCGAAGGGAGCATTGA	126
PRL-R	NM_011169.5	TGGGAGATCCACTTCACAGG	GGCCACAATGATCCACACA	189
MUP3	NM_001039544	GAGCTTTTGTGGAAAACATCACT	TTGTTCACCAATCGCAGTCA	104
ACTB	NM_007393.5	CTGGGACGACATGGAGAAGA	ACCAGAGGCATACAGGGACA	205
HPRT	NM_013556	CAGTCAACGGGGGACATAAA	AGAGGTCCTTTTCACCAGCAA	183
Cyclophilin A	NM_008907	TGGTCTTTGGGAAGGTGAAAG	TGTCCACAGTCGGAAATGGT	109

Supplemental Figure 1

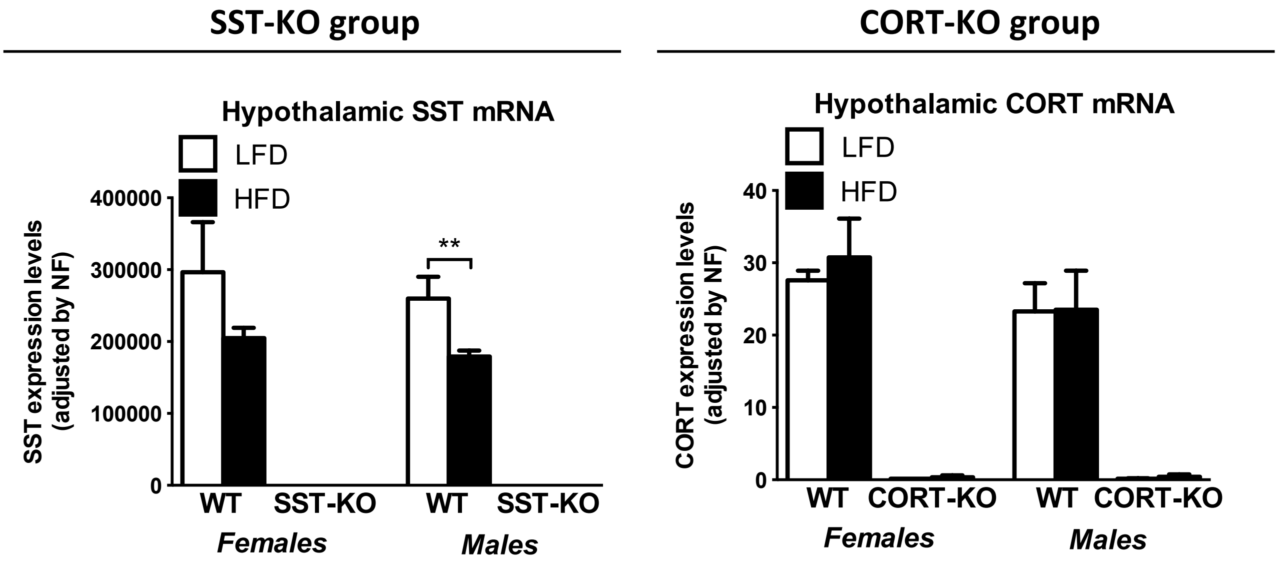

Supplemental Figure 2

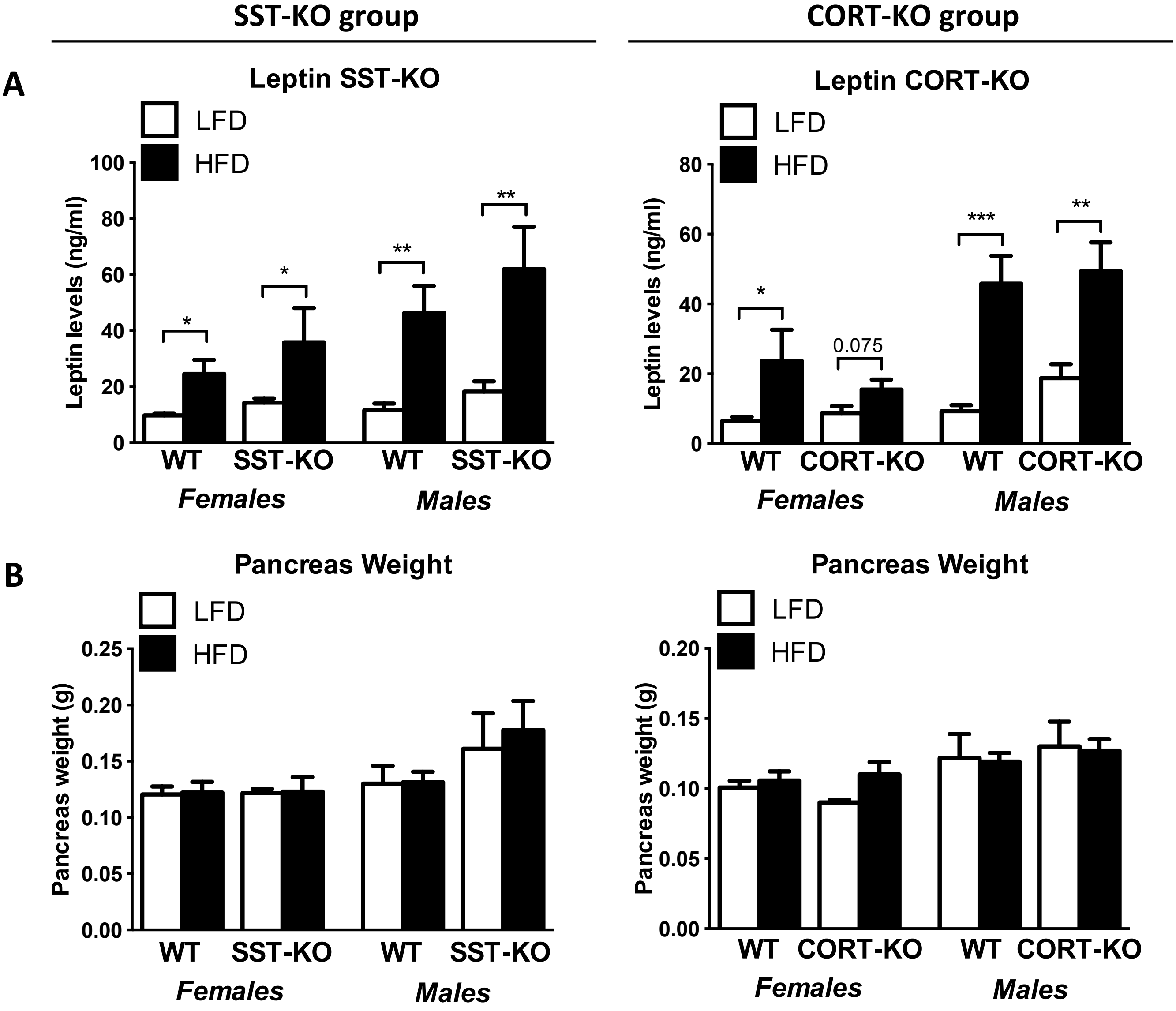

Supplemental Figure 3

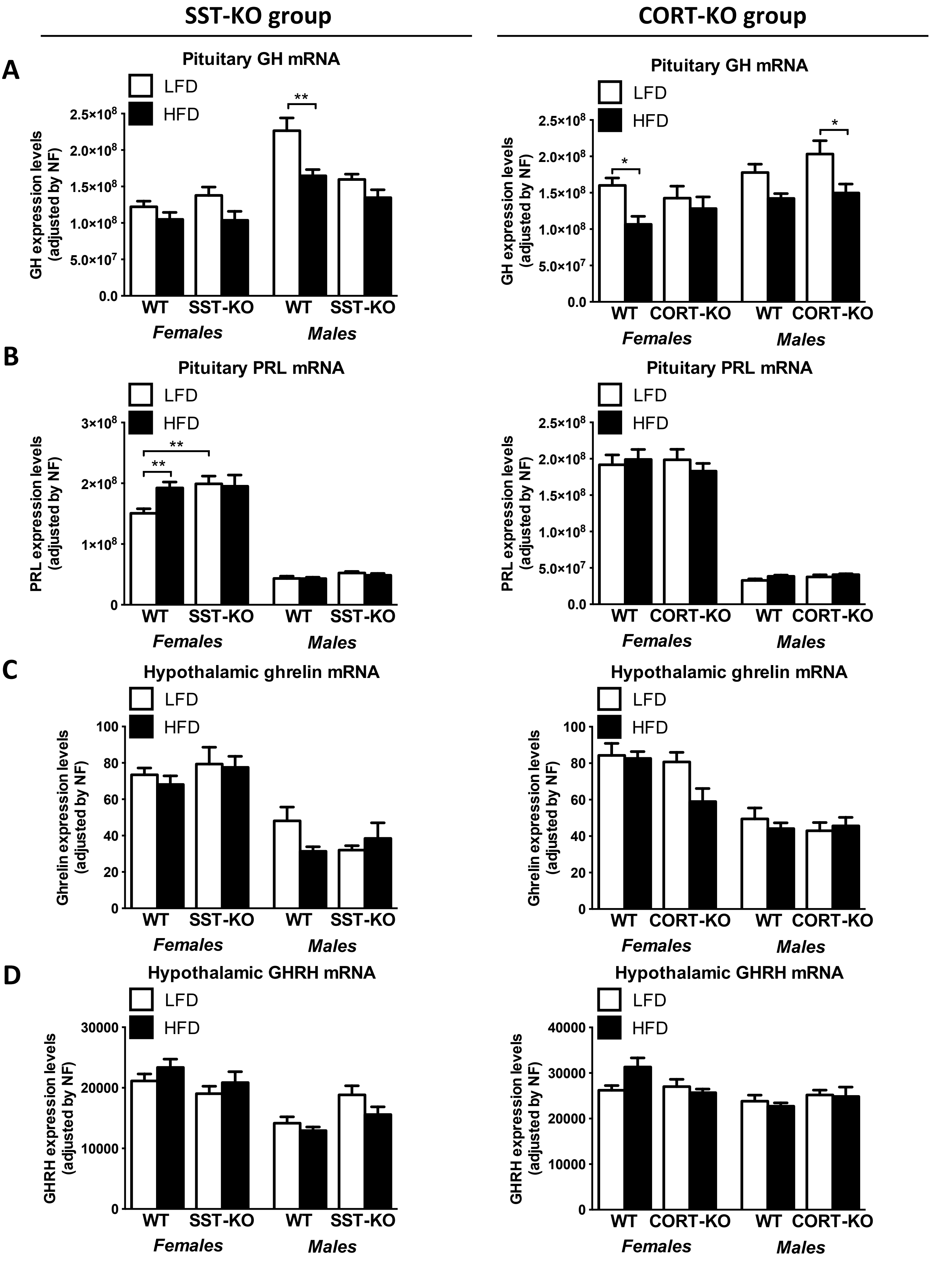

Supplemental Figure 4

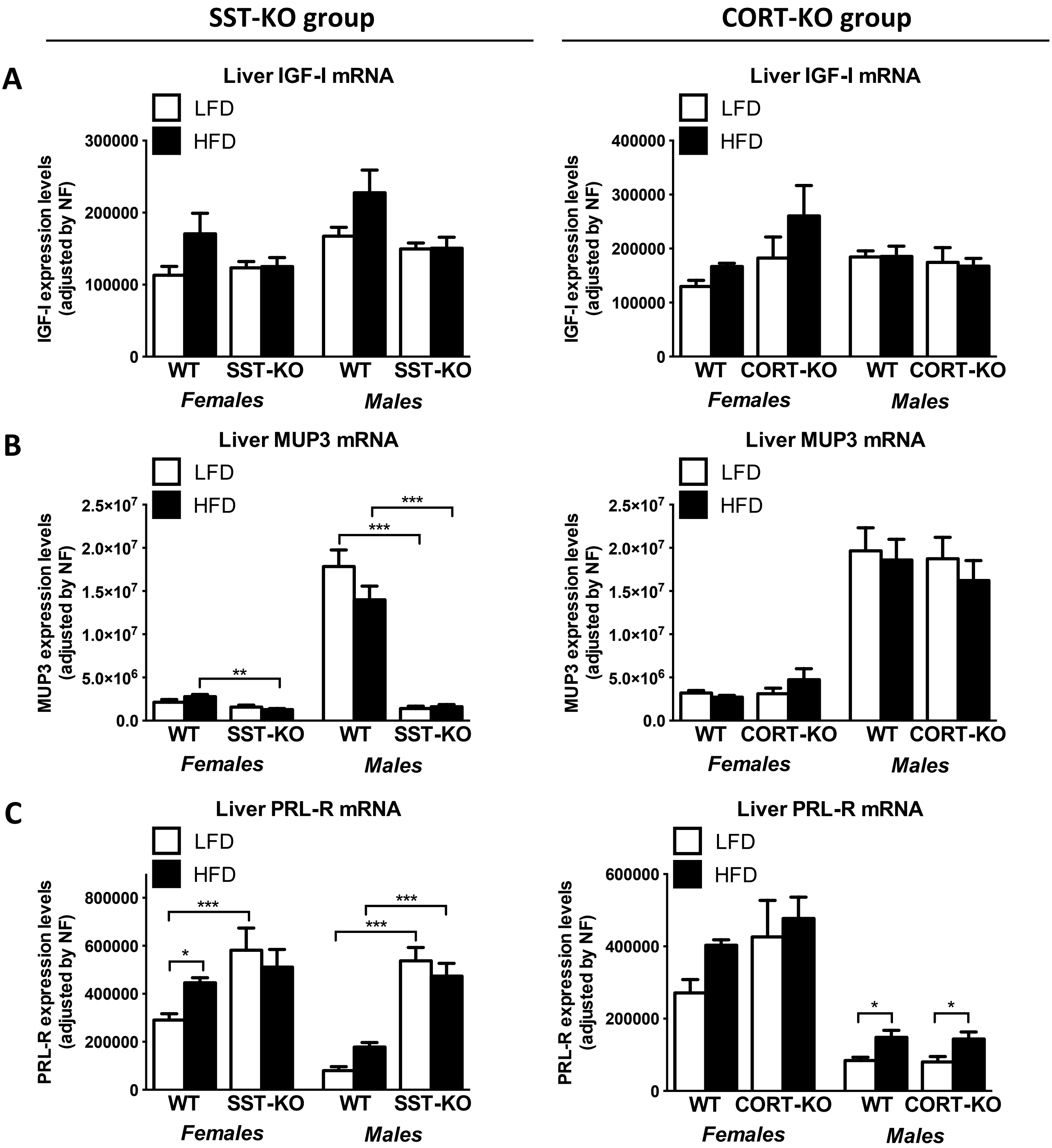

Supplemental Figure 5

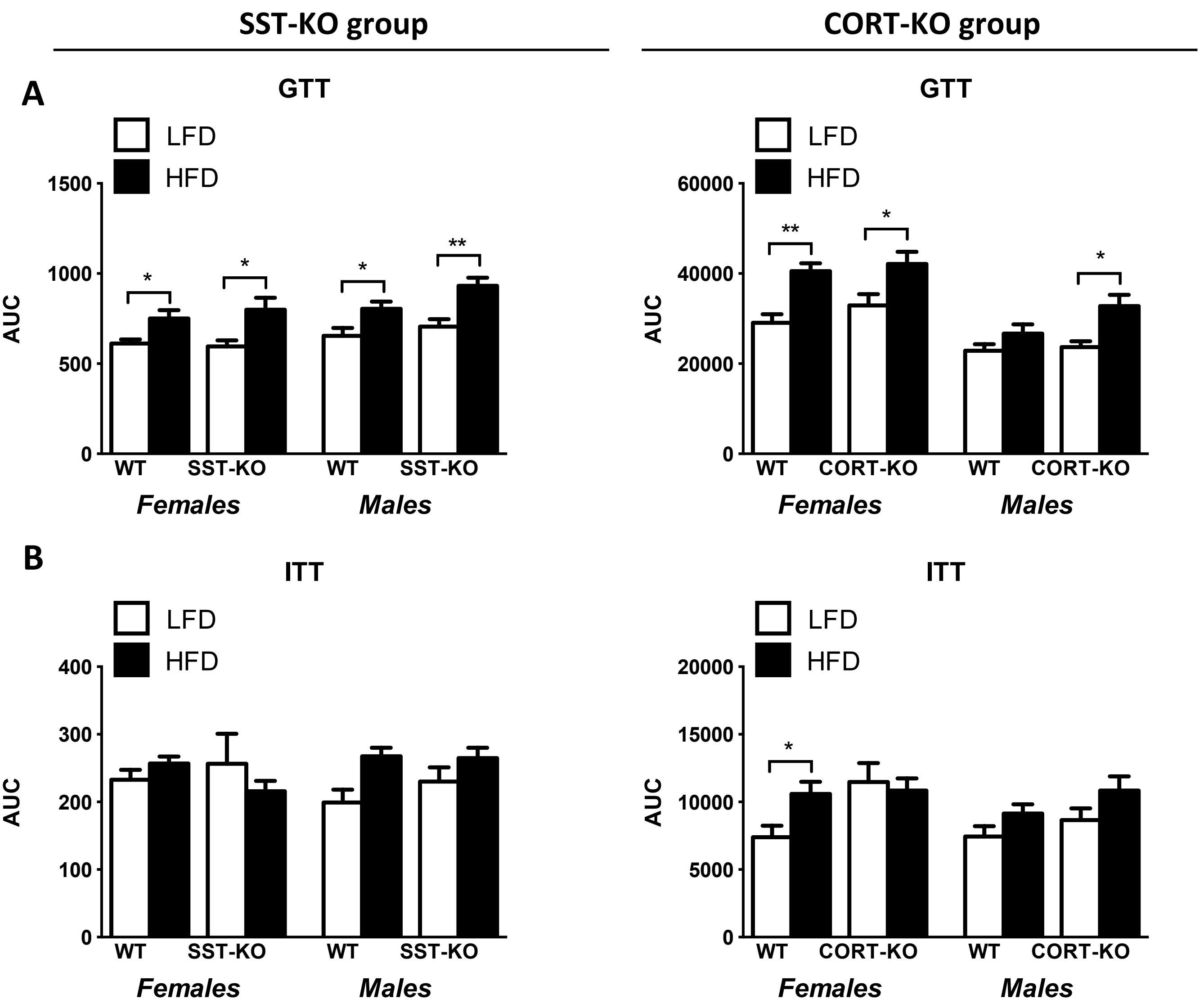

Supplemental Figure 6

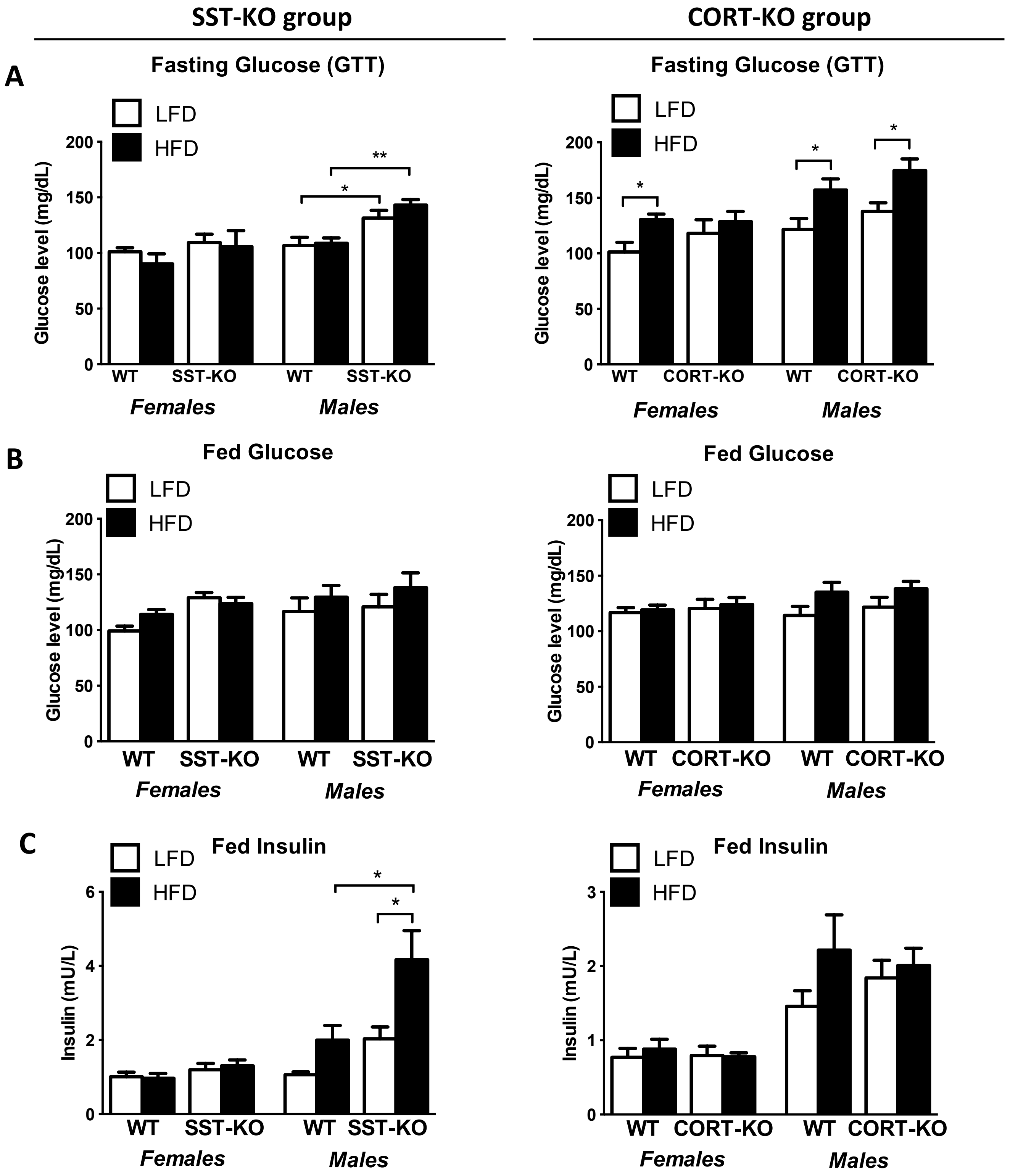

Supplemental Figure 1. Hypothalamic SST and CORT mRNA levels of male and female, LF- and HF-fed, SST-KO, CORT-KO and control mice. qPCR was used to determine hypothalamic mRNA levels of SST on female and male SST-KO and control (WT) mice fed a LF- of a HF-diet and hypothalamic mRNA levels of CORT on female and male CORT-KO and control (WT) mice fed a LF- of a HF-diet. Absolute mRNA levels were adjusted by a normalization factor (NF) determined from the expression of three housekeeping genes (ACTB, HPRT and cyclophilin A). Statistical differences were assessed by two-way ANOVA within genotype and gender followed by Bonferroni post-hoc test [significant differences between LF- and HF-fed mice are indicated by asterisks (**, $p<0.01$). Data represent MEM±SEM of n=6-12 mice per gender, diet and genotype.

Supplemental Figure 2. Leptin levels and pancreas weight of male and female, LF- and HF-fed, SST-KO, CORT-KO and control mice. Plasma leptin levels (A) and pancreas weight (B) of female and male SST-KO, CORT-KO and control (WT) mice fed a LF- of a HF-diet determined at sacrifice (14-16 weeks of diet). Statistical differences were assessed by two-way ANOVA within genotype and gender followed by Bonferroni post-hoc test [significant differences between LF- and HF-fed mice are indicated by asterisks (***, $p<0.001$; **, $p<0.01$; *, $p<0.05$). Data represent MEM±SEM of n=6-12 mice per gender, diet and genotype.

Supplemental Figure 3. Pituitary GH and prolactin (PRL) and hypothalamic ghrelin and GHRH mRNA levels of male and female, LF- and HF-fed, SST-KO, CORT-KO and control mice. qPCR was used to determine mRNA levels of pituitary GH (A) and PRL (B) and hypothalamic ghrelin (C) and GHRH (D) on female and male SST-KO, CORT-KO and control (WT) mice fed a LF- of a HF-diet. Absolute mRNA levels were adjusted by a normalization factor (NF) determined from the expression of three housekeeping genes (ACTB, HPRT and cyclophilin A). Statistical differences were assessed by two-way ANOVA within genotype and gender followed by Bonferroni post-hoc test [significant differences between LF- and HF-fed mice are indicated by asterisks (**, $p<0.01$; *, $p<0.05$). Data represent MEM±SEM of n=6-12 mice per gender, diet and genotype.

Supplemental Figure 4. Hepatic IGF-I, MUP3 and PRL-R mRNA levels of male and female, LF- and HF-fed, SST-KO, CORT-KO and control mice. qPCR was used to determine hepatic mRNA levels of IGF-I (A), MUP3 (B) and PRL-R (C) on female and male SST-KO, CORT-KO and control (WT) mice fed a LF- of a HF-diet. Absolute mRNA levels were adjusted by a normalization factor (NF) determined from the expression of three housekeeping genes (ACTB, HPRT and cyclophilin A). Statistical differences were assessed by two-way ANOVA within genotype and gender followed by Bonferroni post-hoc test [significant differences between LF- and HF-fed mice are indicated by asterisks (***, $p<0.001$; **, $p<0.01$; *, $p<0.05$). Data represent MEM±SEM of n=6-12 mice per gender, diet and genotype.

Supplemental Figure 5. Areas under the curve (AUC) determined from dynamic glucose and insulin tolerance tests of male and female, LF- and HF-fed, SST-KO, CORT-KO and control mice. Statistical differences were assessed by two-way ANOVA within genotype and gender followed by Bonferroni post-hoc test [significant differences between LF- and HF-fed mice are indicated by asterisks (**, $p<0.01$; *, $p<0.05$). Data represent MEM±SEM of n=6-12 mice per gender, diet and genotype.

Supplemental Figure 6. Glucose and insulin levels of male and female, LF- and HF-fed, SST-KO, CORT-KO and control mice. Fasting plasma levels of glucose during the GTT (A) and fed glucose (B) and insulin (C) at sacrifice were determined on female and male SST-KO, CORT-KO and control (WT) mice fed a LF- of a HF-diet. Statistical differences were assessed by two-way ANOVA within genotype and gender followed by Bonferroni post-hoc test [significant differences between LF- and HF-fed mice are indicated by asterisks (**, $p<0.01$; *, $p<0.05$). Data represent MEM±SEM of n=6-12 mice per gender, diet and genotype.
